# How is the human microbiome linked to kidney stones?

**DOI:** 10.3389/fcimb.2025.1602413

**Published:** 2025-06-06

**Authors:** Xin Pei, Miao Liu, Shanshan Yu

**Affiliations:** ^1^ Department of Urology, China-Japan Union Hospital, Jilin University, Changchun, China; ^2^ Department of Cardiovascular Medicine, China-Japan Union Hospital, Jilin University, Changchun, China; ^3^ Department of Anaesthesiology, China-Japan Union Hospital, Jilin University, Changchun, China

**Keywords:** human microbiome, microbiome, kidney stones, immune microenvironment, stone

## Abstract

In recent years, the incidence of kidney stones has continued to rise worldwide, and conventional treatments have limited efficacy in treating stones associated with recurrent or metabolic abnormalities. The microbiome, as the ‘second genome’ of the host, is involved in the development of kidney stones through metabolic regulation, immune homeostasis and inflammatory response. Studies have shown that the urinary microbiome of healthy people is dominated by commensal bacteria such as Lactobacillus and Streptococcus, which maintain microenvironmental homeostasis, whereas patients with renal stones have a significantly reduced diversity of intestinal and urinary microbiomes, with a reduced abundance of oxalic acid-degrading bacteria (e.g., Bifidobacterium oxalicum, Bifidobacterium bifidum), and a possible concentration of pathogenic bacteria (e.g., Proteus mirabilis). The microbiome regulates stone formation through mechanisms such as metabolites (e.g., short-chain fatty acids), changes in urine physicochemical properties (e.g., elevated pH), and imbalances in the inflammatory and immune microenvironments. For example, urease-producing bacteria promote magnesium ammonium phosphate stone formation through the breakdown of urea, whereas dysbiosis of the intestinal flora increases urinary oxalic acid excretion and exacerbates the risk of calcium oxalate stones. Microbiome-based diagnostic markers (e.g., elevated abundance of Aspergillus phylum) and targeted intervention strategies (e.g., probiotic supplementation, faecal bacteria transplantation) show potential for clinical application. However, technical bottlenecks (e.g., sequencing bias in low-biomass samples), mechanistic complexity (e.g., multistrain synergism), and individual heterogeneity remain major challenges for future research. Integration of multi-omics data, development of personalised therapies and interdisciplinary research will be the core directions to decipher the relationship between microbiome and kidney stones.

## 1 Introduction

In recent years, the global incidence of kidney stones has shown a rising trend year by year, and the recrence rate is high, which has become an important burden of urological diseases. Although existing treatments (e.g., pharmacological lithotripsy, extracorporeal lithotripsy) have relieved symptoms to some extent, they have limited efficacy in some patients, especially in cases associated with recurrent stones or metabolic abnormalities (Choi et al., 2023).Traditional therapies focus on physical interventions or chemical lithotripsy, failing to address the metabolic and microenvironmental imbalances that underlie stone formation, and there is an urgent need to explore the pathological mechanisms from a new biological perspective (Gu et al., 2021).The microbiome, as the ‘second genome’ of the host, plays a central role in metabolic regulation, immune homeostasis and inflammatory responses (Shaffer et al., 2019) (Jean-Pierre et al., 2021).Studies have shown that the urinary microbiome (including urinary, intestinal and vaginal commensal flora) is strongly associated with urological diseases, such as cystitis and interstitial nephritis (Choi et al., 2023).The gut microbiome regulates host metabolism through metabolites (e.g. short-chain fatty acids, bile acids) and may influence the concentration of urinary stone-forming substances (e.g. oxalic acid, calcium ions) (Jean-Pierre et al., 2021) (Hertel et al., 2023).In addition, urinary tract pathogens (e.g. E. coli) can promote stone production directly through biofilm formation or inflammatory responses, For example, Escherichia coli promotes CaOx stone formation by enhancing PPK1/flagellin-regulated oxidative damage and inflammation (Choi et al., 2023) (An et al., 2021).These findings suggest that the microbiome may be involved in the development of kidney stones through the ‘gut-kidney axis’ or local colonisation (Gu et al., 2021).Elucidating the mechanism of interaction between the microbiome and kidney stones is expected to lead to breakthroughs in diagnostic and therapeutic strategies. First, microbiome profiles can be used as biomarkers to predict recurrence risk (Xiang et al., 2021).Secondly, targeted modulation of specific flora or their metabolites (e.g. butyric acid, indole derivatives) may inhibit stone nucleation or dissolve existing stones (Hertel et al., 2023);Finally, by intervening in microbiome-host metabolic networks (e.g. oxalic acid degradation, immunomodulation), it may be possible to make the transition from ‘symptomatic’ to ‘etiological’ treatment (Shaffer et al., 2019).This research direction not only expands the knowledge of the pathological mechanisms of kidney stones, but also provides a scientific basis for the development of novel therapies such as microecological agents and personalised dietary interventions. Therefore, there is an urgent need to study the relationship between the human microbiome and kidney stones.

## 2 Microbiome differences between healthy and kidney stone patients

### 2.1 Characterisation of the microbiome of the healthy urinary system

Core urethral and bladder flora (e.g., Lactobacillus, Streptococcus) and their function in maintaining microenvironmental homeostasis in healthy people. In the lower urinary tract of healthy women, Lactobacillus is one of the dominant genera, especially Lactobacillus crispatus and Lactobacillus gasseri (Zheng et al., 2024).In asymptomatic populations, Lactobacillus accounted for more than 15 per cent of the relative abundance of the bladder microbiota. Lactobacilli were also present in the urine microbiome of men, but their abundance was lower than that of women (Xia et al., 2023).Streptococcus is another common group of commensal bacteria, accounting for about 11.9 per cent of the healthy population. Together with Lactobacillus, it maintains flora homeostasis and may inhibit pathogen colonisation competitively (Thomas-White et al., 2018).Corynebacterium, Staphylococcus, Actinomyces and others are also part of the microbiome of a healthy urinary system (Brubaker and Wolfe, 2017).

Lactobacillus reduces urine pH by secreting lactic acid, creating an acidic environment that is not conducive to the growth of pathogens such as E. coli. Lactobacilli also produce bacteriocins and hydrogen peroxide, which directly inhibit the proliferation of pathogens (Zheng et al., 2024).Symbiotic bacteria prevent pathogens from attacking by promoting the secretion of mucin and antimicrobial peptides by the uroepithelial cells, enhancing the mucosal barrier function. Core flora, such as Lactobacillus, regulate the balance of pro-inflammatory and anti-inflammatory responses by activating local immune cells (e.g. dendritic cells) to avoid excessive inflammation (Kawalec and Zwolińska, 2022).

### 2.2 Microbiome changes in patients with kidney stones

**Stone-associated flora enrichment:** Certain species of the genus Aspergillus (e.g. urease-producing bacteria) can produce ammonia by breaking down urea, leading to alkalinisation of the urine and promoting the formation of magnesium ammonium phosphate (guano) (Liu et al., 2021). The formation of these stones is closely associated with urinary tract infections, but there is no direct reference to Aspergillus spp. in the specific evidence, and further inferences need to be made in the context of the microbial urease-producing pathomechanism. The presence of Bacillus oxalicus, a specialised oxalate-degrading bacterium, reduces intestinal oxalate absorption and thus urinary oxalate excretion. Individuals carrying B. oxalans were shown to have a significant 19.5% lower urinary oxalate excretion on a low-calcium/medium-oxalate diet, suggesting that it may reduce the risk of calcium oxalate stones. In addition, animal experiments have shown that B. oxalans promotes intestinal oxalate secretion and further reduces urinary oxalate loading (Jiang et al., 2011). A randomised controlled trial of oral Bacillus oxalicus (Oxabact) in patients with primary hyperoxaluria (PH) did not show a significant improvement in urinary oxalate excretion and may be related to the severity of the abnormalities in oxalate metabolism in the patients or strain adaptation (Hoppe et al., 2011). In addition, oxalobacteria are sensitive to common antibiotics, and prolonged use of antibiotics may lead to a decrease in their colonisation and increase the risk of stones. Therefore, further clinical studies are needed to support the relationship between B. oxalans and the development of kidney stones.

Bifidobacterium lactis (e.g. B. lactis) effectively degrades oxalic acid by expressing oxaloacetyl coenzyme A decarboxylase (Oxc), which has been shown to reduce urinary oxalic acid levels in animal studies (Klimesova et al., 2015).Lactobacilli (e.g. L. acidophilus and S. thermophilus) exhibit oxalic acid degrading activity in *in vitro* and clinical studies, but their effects are influenced by dietary oxalic acid concentration and strain specificity (Karamad et al., 2022). When combined with the intervention, a high-dose Lactobacillus mixture (containing Lactobacillus and Bifidobacterium) reduced urinary oxalic acid excretion from a baseline of 55.5 mg/24h to 28.3 mg/24h in patients with hyperoxaluria, with an effect that lasted up to 1 month after discontinuation of the drug. Zinc ions (Zn²^+^) enhance oxalate decarboxylase (OxDC) activity and protect Lactobacillus from antibiotic damage, further reducing the risk of stone development (Campieri et al., 2001).

**Diversity differences:** Reduced alpha diversity: the alpha diversity of intestinal and urinary microorganisms is significantly reduced in patients with kidney stones and may be related to flora imbalance due to antibiotic use, inflammation, or disturbances in oxalate metabolism. Differences in β-diversity: the composition of the flora (β-diversity) in patients with stones is significantly different from that of the healthy population, e.g., there is a reduction in the abundance of oxalic acid-degrading organisms (e.g., oxalobacteria, bifidobacteria), while potentially pathogenic bacteria (e.g., metaplastic organisms) may be enriched. Impact of antibiotics: Antibiotic use can disrupt oxalate-degrading flora and increase stone risk. For example, the colonisation rate of B. oxalans is significantly lower in individuals on long-term broad-spectrum antibiotic use (Campieri et al., 2001) (Liu et al., 2020). That’s why long-term antibiotic use may worsen the risk of kidney stones, and some academics have shown in studies that it may be worse in children (Tasian et al., 2018) (Ferraro et al., 2019).

### 2.3 Stone type and microbiome specificity

Calcium oxalate stones are associated with an imbalance in the intestinal oxalate metabolising flora: specific intestinal bacteria such as Oxalobacter formigenes, Lactobacillus and Bifidobacterium are effective in degrading oxalic acid, reducing intestinal absorption and urinary excretion of oxalic acid (Razi et al., 2024).For example, O. formigenes degrades about 51 per cent of dietary oxalic acid on a daily basis by breaking it down into formic acid and carbon dioxide. Lack of these bacteria (e.g., due to prolonged antibiotic use) can lead to increased oxalic acid absorption and elevated urinary oxalic acid levels, which can promote calcium oxalate stone formation (Miller et al., 2016) (Wallace et al., 2023). Potential of probiotic interventions: experiments have shown that supplementation with high concentrations of lactic acid bacteria (e.g. L. acidophilus, S. thermophilus) significantly reduces urinary oxalic acid concentration and inhibits stone formation (Sasikumar et al., 2014). Recombinant lactobacilli even secrete oxalate decarboxylase, which directly degrades oxalic acid. Faecal microbial transplantation (FMT) has also been shown to regulate urinary oxalic acid excretion and restore flora balance (Miller et al., 2016). Diet and flora interact: low-calcium diets lead to an increase in intestinal free oxalic acid, which promotes absorption, while calcium supplements reduce its bioavailability by binding to oxalic acid (Ticinesi et al., 2020) (D’Alessandro et al., 2019). Diets high in animal protein or salt reduce beneficial bacteria (e.g. Bifidobacterium bifidum, Lactobacillus lactis) while increasing the proportion of bile-tolerant bacteria, further disrupting the balance of oxalate metabolism (Wallace et al., 2023).

The formation of infected stones (e.g., magnesium ammonium phosphate stones) is directly related to the colonisation and biofilm formation of uropathogenic bacteria. Urease activity of pathogenic bacteria: Urease-degrading bacteria such as Proteus, Klebsiella and Pseudomonas can break down urea into ammonia and carbon dioxide by secreting urease, leading to alkalinisation of the urine (pH >7.0) and promoting the crystallisation of magnesium ammonium phosphate and carbonate apatite (Wallace et al., 2023) (Jung and Lee, 2021). Biofilm promotion: these pathogenic bacteria can form biofilms on the surface of the urinary epithelium or stones, protecting themselves from host immunity and antibiotic attack, as well as providing a substrate for crystal aggregation (Jung and Lee, 2021). Calcium oxalate deposits (e.g. Randall’s plaques) may serve as attachment sites for bacteria, and pathogenic bacteria such as E. coli may selectively aggregate around the crystals, increasing the risk of infection (Stepanova, 2021). Bacterial imbalance and risk of infection: In patients with recurrent pyelonephritis, an imbalance of the intestinal flora (e.g., accumulation of toxins such as lipopolysaccharides) may exacerbate urinary inflammation through the ‘gut-kidney axis’ and promote stone formation. Urinary microbiome diversity is lower in patients with urinary stones, and the abundance of specific pathogenic bacteria (e.g. Escherichia coli) is increased (Jung and Lee, 2021) (Stepanova, 2021).

## 3 Mechanisms of microbiome regulation of kidney stone formation

### 3.1 Metabolite regulation

Oxalic acid and uric acid metabolism: intestinal flora directly influence urinary supersaturation by degrading oxalic acid (e.g. Oxalobacter formigenes) or synthesising uric acid (e.g. Purine metabolising bacteria). For example, specific flora such as Bacteroides are associated with low uric acid levels, whereas a decrease in Coprococcus may lead to an imbalance in uric acid metabolism (Lin et al., 2018). In addition, probiotics such as Bifidobacterium and Faecalibacterium may regulate uric acid levels through compensatory proliferation (Wang et al., 2022) (Agudelo and Miller, 2021). In addition, other oxalate-degrading bacteria (e.g. certain lactobacilli) inhibit crystallisation by producing Formyl-CoA transferase and Oxalyl-CoA decarboxylase, which convert oxalate to substances such as pyruvate (Wang et al., 2021).

Short-chain fatty acids (SCFAs) inhibit stone formation by regulating host metabolism. SCFAs (e.g., acetic acid, propionic acid) modulate the renal immune microenvironment by activating the GPR43 receptor. This is manifested by increasing CX3CR1+CD24-macrophage frequency and decreasing GR1+ neutrophil infiltration, thereby inhibiting calcium oxalate crystal deposition. This process is dependent on the gut microbiota and GPR43 knockdown impairs the protective effect of SCFAs (Jin et al., 2021).

### 3.2 Changes in the physical and chemical properties of urine

Urease-producing bacteria (e.g., Aspergillus spp.) may elevate urine pH and promote phosphate crystallisation by breaking down urea. However, evidence suggests that gut flora metabolites (e.g., citric acid) may directly regulate urine composition by inhibiting calcium oxalate crystallisation. For example, Bacteroides is associated with oxalate metabolism and may indirectly influence urine supersaturation (Lin et al., 2018).

The effect of microbial metabolites (e.g., citric acid) on crystallisation inhibitory factors: Microbial metabolites such as citric acid may enhance urinary inhibition of crystal formation, and dysbiosis may lead to a decrease in such metabolites, exacerbating stone risk (Lin et al., 2018).

### 3.3 Inflammation and the immune microenvironment

Chronic inflammation promotes stone formation: pathogenic bacteria induce the release of pro-inflammatory factors such as IL-6 and TNF-α through activation of the TLR4/NF-κB pathway, promoting stone adhesion and growth (Jin et al., 2021). For example, increased neutrophil infiltration was significantly associated with calcium oxalate deposition (Dong et al., 2023).

Probiotics such as Lactobacillus inhibit the inflammatory response by enhancing mucosal barrier function and upregulating the expression of tight junction proteins (e.g. ZO-1, occludin) (Zhou et al., 2021). At the same time, Lactobacillus metabolites (e.g. lactic acid) down-regulate c-Myc signalling, reducing nucleotide supply and inhibiting pathogen replication (Jin et al., 2021). In addition, Bifidobacterium bifidum and others maintain immune homeostasis by regulating Th1/Th2 balance and increasing anti-inflammatory factors such as IL-10 (Wang et al., 2022) (Zhou et al., 2021).

## 4 The potential of the microbiome in the diagnosis and treatment of kidney stones

### 4.1 Diagnostic marker

Recent studies have found that the abundance of Aspergillus phylum (e.g., Proteobacteria) is significantly higher in the urinary microbiome of patients with kidney stones, whereas the thick-walled phylum predominates in the healthy population. In addition, urinary metabolomics showed that abnormal levels of uric acid, oxalic acid, and purine metabolites were positively correlated with the abundance of urease-producing bacteria (e.g., Streptococcus) in the urinary microbiome of patients with kidney stones, suggesting that the combination of microbial-metabolite markers may improve the diagnostic specificity (Gao et al., 2022).

Significantly lower abundance of SCFAs-producing flora (e.g. Lachnospiraceae, Roseburia) and reduced faecal butyric acid content in the gut of patients with renal stones and exogenous supplementation of SCFAs inhibits renal calcium oxalate crystal formation (Cao et al., 2024) (Stanford et al., 2020). Potential new non-invasive diagnostic tool by detecting faecal SCFAs levels in combination with gut flora analysis (Cao et al., 2024).

### 4.2 Prognostic assessment

Flora diversity and inflammatory state: patients with kidney stones have an increase in pro-inflammatory bacteria (e.g. Megamonas, Escherichia) and a decrease in anti-inflammatory bacteria (e.g. Faecalibacterium prausnitzii) in the gut, an imbalance that may exacerbate renal inflammation and fibrosis through activation of the macrophage mTOR pathway, and a decrease in the flora diversity index correlates with worsening renal function indicators (e.g., Cystatin C) correlated with worsening (Stanford et al., 2020).

The controversial role of oxalate-degrading bacteria: although Oxalobacter formigenes abundance has been correlated with oxalic acid metabolism, some studies have pointed to a controversial association with stone recurrence, which may be subject to host genetic or dietary interference. A comprehensive assessment in conjunction with host metabolic phenotypes (e.g., urinary oxalate excretion) is required (Stanford et al., 2020).

### 4.3 Treatment strategy

**Probiotics/Prebiotics Precision Intervention:** Oxalobacter formigenes supplementation reduces intestinal oxalic acid absorption, while prebiotics containing oligogalactose (GOS) and polydextrose (PDX) selectively stimulate the proliferation of beneficial bacteria, such as Bacteroides uniformis, to improve oxalic acid metabolism (Sonnenburg and Fischbach, 2011) (Thompson et al., 2024). Combined use of probiotics and prebiotics may enhance efficacy (Sonnenburg and Fischbach, 2011).

**Metabolite modulation:** potassium sodium citrate (PSHC) treatment significantly increases patients’ faecal butyric acid levels and inhibits stone formation (Cao et al., 2024). Targeting metabolic pathways of SCFAs (e.g. acetate/propionate synthesis) may become a new direction (Stanford et al., 2020).

**Antibiotic-targeted therapy:** inhibition of urease-producing pathogens (e.g. Enterobacteriaceae) reduces infected stones, but broad-spectrum antibiotics-induced dysbiosis needs to be avoided (Stanford et al., 2020). Future combination with faecal mushroom transplantation (FMT) or specific phage therapies may be safer, further accreditation required.

**Dietary-microbiological co-regulation:** a low oxalate diet combined with probiotics can regulate sulphur-metabolising flora and reduce the production of stone-promoting metabolites such as hydrogen sulphide. In addition, the Mediterranean diet (rich in dietary fibre) promotes the proliferation of SCFAs-producing bacteria and reduces the risk of stone recurrence (Cao et al., 2024) (Gradisteanu Pircalabioru et al., 2022).

There are some controversies and challenges here that need to be further investigated in the future. Heterogeneity of the bacterial flora: differences in the microbiome characteristics of different stone types (e.g. uric acid stones vs. calcium oxalate stones) need to be investigated by typology (Gao et al., 2022). Causal mechanisms are unknown: longitudinal cohort validation is needed to determine whether microbiome changes are stone-causing or secondary outcomes (Stanford et al., 2020) (Gradisteanu Pircalabioru et al., 2022). Individualised treatment: precise interventions need to be designed in the context of the host’s genetic background (e.g. polymorphisms in the SLC26A6 oxalate transporter gene) (Sonnenburg and Fischbach, 2011) (Thompson et al., 2024).

## 5 Challenges and future directions

### 5.1 Technological bottleneck

Inadequate sequencing accuracy for low-biomass samples: Microbiome analysis of low-biomass samples such as urine is vulnerable to contamination and amplification bias. For example, second-generation sequencing technologies (e.g. Illumina) are high throughput and low cost, but rely on library amplification, which can lead to loss or bias of information for low abundance species (Xianchun et al., 2016). In addition, the PCR amplification process may preferentially amplify high-abundance flora, masking the role of key low-abundance microorganisms (e.g., oxalate-degrading bacteria). Evidence in favour: It was noted that gut microbial diversity was significantly lower in stone patients than in healthy individuals (Chao1 index: 1460 ± 363 vs. 1658 ± 297), which may be related to the lack of sensitivity of sequencing technologies to low-abundance species (Ticinesi et al., 2018). Insufficient standardisation of sample handling: Sample collection, preservation and DNA extraction methods varied considerably across studies, affecting the comparability of results. For example, the freezing time of faecal samples may affect the microbial activity and thus the detection of genes related to oxalate metabolism (Kim et al., 2021).

### 5.2 Limitations of mechanistic studies

Differences between animal models and humans: Existing studies have relied on rodent models, but their gut microbiological composition and metabolic pathways differ significantly from those of humans. For example, Oxalobacter formigenes are highly abundant in the mouse gut, but only partially colonise humans, and the association with stone risk is inconsistent (Ticinesi et al., 2018) (Hill and Sayer, 2019). Humanised mouse or organoid models need to be developed to more realistically simulate ‘gut-kidney axis’ interactions (Kim et al., 2021).

Complexity of microbial functional networks: oxalic acid metabolism is not only dependent on a single strain (e.g. Oxalobacter), but also involves multispecies synergies (e.g. Lactobacillus, Bifidobacterium, etc.) and host metabolic pathways (e.g. Vitamin B6 regulates oxalic acid synthesis) (Crivelli et al., 2021) (Chmiel and Stuivenberg, 2023). Existing studies have focused on a single bacterial group and lacked a holistic analysis of the functional network.

### 5.3 Clinical translation difficulties

Heterogeneity of microbiological markers: Large-scale cohort studies have found ethnic and geographic differences in microbiological markers in stone patients. For example, reduced abundance of Faecalibacterium and Dorea was associated with stones in studies of Italian populations, but may be confounded by diet (e.g., high oxalic acid intake) or antibiotic use in other regions (Ticinesi et al., 2018). Multi-centre studies are needed to control for confounding factors (Kim et al., 2021).

Dietary and pharmacological interference: high-calcium diets may increase stone risk but also inhibit oxalate uptake, the net effect of which needs to be assessed in the context of individual microbiome profiling (Crivelli et al., 2021). In addition, antibiotic abuse may destroy the oxalate-degrading flora and indirectly promote stone formation (Kim et al., 2021).

### 5.4 Future direction

**Multi-omics integration:** Combining macro-genomic (flora composition), metabolomic (oxalic acid, short-chain fatty acids, etc.) and proteomic (host enzyme activity) data, microbe-host metabolic interactions can be comprehensively analysed. For example, reduced expression of oxalate-degrading genes (e.g. oxc, frc) in the gut of stone patients was found to be directly associated with increased urinary oxalate excretion (r=-0.87, p=0.002) (Ticinesi et al., 2018).

**Personalised interventions:** precision probiotic approach: formulas tailored to the patient’s microbiological profile, e.g. supplementation with Oxalobacter or butyric acid-producing bacteria (Faecalibacterium) to enhance oxalic acid degradation (Ticinesi et al., 2018) (Hill and Sayer, 2019). Faecal bacteria transplantation (FMT): reduces the risk of stone recurrence by restoring the balance of intestinal flora, but requires rigorous donor selection and optimisation of transplantation protocols (Kim et al., 2021).

**Interdisciplinary research:** Investigating the bidirectional effects of intestinal barrier function (e.g., tight junction proteins), immunomodulation (e.g., the pro-inflammatory factor IL-6), and environmental factors (e.g., antibiotics, heavy metals) on the ‘gut-kidney axis’. For example, short-chain fatty acids (SCFAs) may play a protective role by inhibiting renal tubular oxalic acid crystal deposition (Kim et al., 2021) (Mocanu et al., 2023).

## 6 Conclusion

The microbiome plays a key role in the formation, recurrence and treatment of kidney stones. The microbiome profiles of healthy and stone-forming patients differed significantly, with a reduction in oxalate-degrading organisms and an enrichment of pathogenic organisms being the central hallmarks. The microbiome influences stone formation through metabolic regulation, inflammatory response, and alteration of the urinary microenvironment, particularly through the ‘gut-kidney axis’. Intervention strategies targeting the microbiome (e.g., probiotics, metabolite modulation) provide new ideas to reduce stone risk, but technical limitations and individual heterogeneity need to be overcome. Future studies need to incorporate multi-omics techniques to analyse microbe-host interaction networks, develop precise diagnostic protocols, and validate the causality of microbiome changes through longitudinal cohorts. The development of this field is expected to shift the treatment of kidney stones from ‘symptomatic’ to ‘etiological’ and provide more efficient and personalised management strategies for patients.
